# Use of Market Research Data by State Chronic Disease Programs, Illinois, 2012–2014

**DOI:** 10.5888/pcd11.140268

**Published:** 2014-09-25

**Authors:** Nancy L. Amerson, Benjamin S. Arbise, Nora K. Kelly, Elizabeth Traore

**Affiliations:** Author Affiliations: Benjamin S. Arbise, Nora K. Kelly, Illinois Department of Public Health, Office of Health Promotion, Division of Chronic Disease Prevention and Control, Springfield, Illinois; Elizabeth Traore, Directors of Health Promotion and Education, Washington, DC.

## Abstract

Market research data complement traditional epidemiologic data by allowing users to examine health behavior and patterns by census block or census tract. Market research data can identify products and behaviors that align or do not align with public health program goals. Illinois is a recipient of an award from the Directors of Health Promotion and Education to use industry market research data collected by The Nielsen Company for public health purposes. Illinois creates customized community profiles using market research data on tobacco use characteristics to describe the demographics, habits, and media preferences of smokers in certain locations. Local agencies use profiles to plan and target marketing initiatives, reach disparate groups within overall community populations, and restructure program objectives and policy initiatives. Local market research data provide detailed information on the characteristics of smokers, allowing Illinois communities to design public health programs without having to collect data on their own.

## Market Research and Public Health

Most readily available chronic disease data sets in Illinois (eg, state mortality data and data from the Behavioral Risk Factor Surveillance System) are released at the county or state level. However, communities need community-level public health data. Even county data are not specific enough for developing targeted interventions in communities. Market research data offer a way to examine health behavior and patterns in small localities; these data allow users to examine communities by census block or census tract. Marketing data complement traditional epidemiologic data (eg, national and state survey data, mortality data, hospitalization data, program data). Using a combination of data types enhances a program’s ability to define the target population and create a tailored and constructive intervention.

Market research is the process of collecting and analyzing data to help industries make decisions about their products and services (1). These data can be used by public health programs to 1) identify population segments for targeted messages; 2) identify population segments that differ from one another in interests, lifestyle, or media habits; and 3) optimize use of media venues (2). Market research data identify products and behaviors that align or do not align with program goals. These data have been used at various stages of public health campaigns — from development to implementation. Although until recently little research was published on public health initiatives that use market research data (3), the use of private market research firms by local and national organizations for health-related purposes is increasing (4). One study used a combination of community-based participatory research and community-based prevention marketing (marketing principles for public health interventions) to design a health-promotion intervention in Sarasota County, Florida (5). Community-based prevention marketing was also used to develop and implement physical activity interventions for middle-aged women (6) and for children and adolescents (7), but investigators in both of these studies conducted their own market research surveys. Another study used geographic information systems (GIS) data and marketing data to investigate an environmentally based disease in a neighborhood in greater Tucson, Arizona (8).

One efficient marketing method of studying characteristics of people in a population of interest is through segmentation. Segmentation is “a process of looking at the audience or ‘market’ and seeking to identify distinct, manageable subgroups (segments) that may have similar needs, attitudes, or behaviors” (8). The goal of segmentation is to create population subgroups in which all members are as similar to one another and as different from others as possible. Segmentation would improve public health researchers’ understanding of the target population by providing insight that is then used to develop and implement public health programs. Market research provides a perspective on the target population’s various viewpoints, such as political beliefs, lifestyles, psychosocial characteristics, personality traits, consumer patterns, and media habits (9).

## Market Research Data Sets Used by Illinois Department of Public Health

Market research data include proprietary and public information and are available, with individual identifying information removed, through licensing and contractual agreements (9). Several companies collect and analyze market research data, including The Nielsen Company (10), Audit Bureau of Circulations (11), CMR Services (12), Environmental Systems Research Institute (13), Market Research Insight (14), Marketing Performance Group (15), Marshall Marketing (16), and SQAD (17).

The Illinois Department of Public Health (IDPH) has a grant from the Directors of Health Promotion and Education (DHPE) that allows access to 6 Nielsen Company data sets. Nielsen is a leader in market research, and the data provided to IDPH are the same data provided to Fortune 500 companies. Nielsen collects some data itself and purchases other sets of data. IDPH has access to the following Nielsen data sets:

**Nielsen Pop-Facts Premier.** This data set provides current-year estimates and 5-year projections for demographic data based on data from the US Census and the American Community Survey (18).

**PRIZM segmentation system.** The system classifies every household in the United States into 1 of 66 consumer segments on the basis of purchasing and media behavior across a range of categories (eg, communications, media usage). Each segment has a unique demographic description based on income, age, presence of children in the household, home ownership, employment, education, and race and ethnicity (19).

**Mediamark Research and Intelligence (MRI) PRIZM Profiles.** MRI profiles are created from the MRI Survey of the American Consumer, an ongoing, comprehensive study of US adults (aged 18 or older) and 24 categories (eg, demographics, product usage, media exposure). More than 2,400 profiles are built using 2 years of data (approximately 50,000 respondents). Nielsen annually evaluates and updates the profiles, which are weighted on the basis of current-year PRIZM household distributions (20).

**Nielsen Consumer Buying Power.** This data set has geographic estimates of annual consumer expenditures for more than 350 household expenditure items. These data are used to identify the potential demand of consumers for products and services by geographic area regardless of where consumers make their purchase (21). The database was developed using the Consumer Expenditure Survey conducted by the US Bureau of Labor Statistics (22).

**Nielsen Retail Market Power.** This data set has data on consumer expenditures and retail sales for various retail store types and merchandise line items. The database was developed using the Consumer Expenditure Survey and the Census of Retail and Wholesale Trade, conducted by the US Census Bureau (23).

**Nielsen Business-Facts. **This is a comprehensive database of US business and employee counts. It contains data on several characteristics, including contact names, locations, addresses, number of employees, annual sales, and North American Industry Classification System codes, for more than 15 million business locations (24). Infogroup is the company that collects and maintains the data from which this data set is built (25).

## How Illinois Tobacco-Free Communities Use Market Research Data: Creation of a Customized Community Profile

IDPH funds programs at 94 local health departments through the Illinois Tobacco-Free Communities (ITFC) grant for tobacco use prevention and control activities, which include using media to advertise the Illinois Tobacco Quitline (ITQL). ITFC grantees can request a customized community profile to guide planning for programs to promote cessation among young people and adults and to identify and eliminate disparities related to tobacco use and its effects among various population groups. Marketing data provided detailed information to local health departments to guide their decisions about media campaigns and how to spend scarce media dollars for the ITQL. Most local health departments have only county data on the prevalence of tobacco use. Market research data allows local health departments to collect and analyze data on smokers (defined as those who smoked any amount in the previous week), heavy smokers (defined as those who smoke 7 or more packs of cigarettes per week), and smokeless tobacco users in their own communities.

Customized community profiles are created to locate the target population (whether in a county or city) and to describe its demographics, habits, and media preferences. The profile includes maps, data interpretation, and recommendations. The local health department is expected to report to IDPH on how the data were used within 90 days after receiving a customized profile.

One purpose of the customized community profile is to identify populations of heavy smokers. This is achieved by identifying PRIZM segments that are likely to include people who smoke. Fifteen of 54 PRIZM segments in Sangamon County, Illinois, for example, are likely to have people who are heavy smokers. Nearly a quarter of heavy smokers in the county are in just 5 PRIZM segments. PRIZM segment number 53 has an estimated 993 heavy smokers, who account for nearly 7% of heavy smokers in the county (Table 1).

**Table 1:** The Nielsen Company PRIZM Segments in Sangamon County, Illinois, 2014^a^

Segment Identification No.	No. of Smokers in Segment	Heavy Smokers^b^ in County, %^c^	No. of Heavy Smokers per 100 Households in County
53	993	6.8	33.4
47	873	6.0	35.5
32	646	4.4	23.7
35	585	4.2	22.2
33	530	3.6	26.5

a A segment is a subpopulation of a given area that has similar characteristics and preferences (ie, it is a demographic unit, not a geographic unit) and was defined in this study by using market research data from The Nielsen Company (10).

b Heavy smokers defined as those who smoke 7 or more packs of cigarettes per week.

c Number of heavy smokers in segment divided by number of heavy smokers in county.

Once the PRIZM segments are identified, Nielsen software is used to pinpoint high concentrations of those segments by zip code or census tract (Figure). Census tracts generally have a population size between 1,200 and 8,000 people (26). The primary purpose of census tracts is to provide a stable set of geographic units for the presentation of statistical data; the ideal size for the presentation of statistical data is 4,000 people (26). Depending on the location, census tract maps can help tobacco use prevention and control programs to better target tobacco program activities in their communities.

**Figure:**
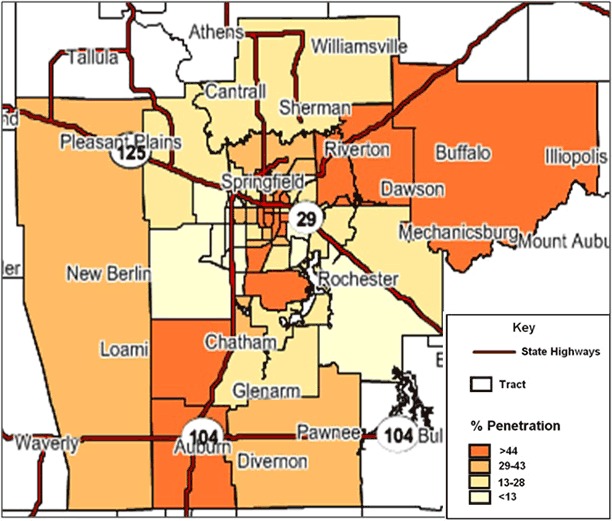
Percentage of heavy smokers in Sangamon County, Illinois, by census tract, 2014. Source of data: Nielsen Pop-Facts, 2014 (18).

Next, Nielsen ConsumerPoint software is used to identify where heavy smokers in a targeted area (eg, Sangamon County) shop, their preferred media outlets (ie, newspapers, magazines, radio, Internet, or television), how frequently they use these media outlets, and their demographic characteristics (eg, age, annual household income, home ownership [eg, rent vs own, size of apartment, value of home], employment, race/ethnicity, household composition). Table 2 is an example of a table provided in the community profile; it shows the media preferences of heavy smokers in Sangamon County and their level of media usage; radio and the Internet are the most used media avenues among heavy smokers. Table 3 is another example of the kind of demographic information in the customized community profile; it shows the types of occupations of heavy smokers and the industries that employ them.

**Table 2:** Media Preferences Among Heavy Smokers in Sangamon County, Illinois, 2014^a^

Type of Media	Level of Media Usage^b^	Average Media Usage	No. of Heavy Smokers^c^ per 100 Households in County
Radio	Very high	26 h/wk	41.0
Total television	Very light	0–9 h/wk	40.6
Daytime primetime television	Very light	0–1 h/wk	40.2
Newspaper	Light	1–7 newspapers/28 d^d^	39.1
Magazine	Very light	0–1 magazines/mo	38.6
Internet	Very high	≥56 times used/mo	38.3

a Source of data: Nielsen PRIZM, 2014 (19).

b Data were grouped into the following quintiles of usage: very high, high, moderate, light, and very light.

c Heavy smokers defined as those who smoke 7 or more packs of cigarettes per week.

d Number of newspapers read in an average 28-day period developed from a weighted average of the number of daily newspapers read in a week (weighted by 4) and the number of Sunday papers read in 4 weeks (weighted by 1), based on the number of issues of newspapers respondent reported reading for each of the 2 periods.

**Table 3:** Employment Profile of Heavy Smokers in Sangamon County, Illinois, 2014^a^

Employment Profile^b^	No. of Heavy Smokers^c^	No. of Heavy Smokers per 100 Households in County
**Occupation**		
Blue-collar–type jobs	3,148	21.6
Service and farms	2,911	20.0
Office/administration support	2,043	14.0
Food preparation/serving	945	6.5
**Industry**		
Retail trade	1,770	12.2
Total manufacturing	1,563	10.7
Educational services	1,432	9.8
Accommodations/food	1,162	8.0
Construction	940	6.5

a Source of data: Nielsen Business-Facts, 2014 (24,25).

b Among the civilian population aged 16 years or older.

c Heavy smokers defined as those who smoke 7 or more packs of cigarettes per week.

## Feedback From Illinois Tobacco-Free Communities

Since November 2012, 40 local health departments in Illinois have received a customized community profile on tobacco use, and 92% of them have sent feedback reports within 90 days of receipt*.* Data from the feedback reports indicate that the community profiles are used for program planning, especially for designing media plans precisely tailored to the preferences of smokers in the community. Instead of conducting county-wide marketing campaigns, local health departments target smokers by zip code and census tract. The community profiles are also used to plan marketing initiatives, to reach disparate populations, to restructure program objectives and policy initiatives, and to assist with enforcing policies.

The Kane County Health Department said this about their community profile: “The tobacco report has definitely influenced our program directions. Instead of targeting the whole county in our outreach we will be targeting the identified communities, namely Aurora (all zip codes), Elgin (60123), and Carpentersville (60110). This report helped us in focusing our media plan in certain segments of the population, as opposed to the whole community in the county.”

Another recipient of a community profile created a coalition to engage the 4 communities identified by the research in discussing policies, enhancing cessation services, and reaching disparate populations in each community. Additionally, marketing funds were reallocated, and programs were restructured to align with the market research findings. The new structure allowed staff to refocus time, energy, programs, and the media budget for the 4 communities.

## Discussion

Studying consumer lifestyle and behavior provides insight into what members of the population are like, what they do, where they are located, and how to reach them. These data can be accessed through collaboration with other agencies or directly from a market research firm. Data can also be collected through private contracts to prepare customized reports.

Illinois and other DHPE grantees used market research data to improve tobacco quitline use; identify the best audiences to target with smoking cessation and prevention advertisements; examine the food environment, including the location of food deserts, the density of fast food restaurants, and the modified Retail Food Environment Index; target health promotion strategies based on life stage; engage the private sector in developing wellness activities in the community; and identify high-risk areas for targeting chronic diseases.

Barriers to use of market research include the costs of obtaining the proprietary software and data sets. The Nielsen Company market research data accessed by local health departments in Illinois through DHPE costs an average $85,000 per year. State health departments may have difficulty purchasing these data because of a lack of resources, but given the amount and versatility of the data, the cost compares well with the cost of other data sets typically accessed by state health departments (eg, hospital discharge data, emergency department discharge data). Public health agencies that purchase market research data can use the results of analysis in applications for new funding opportunities and allocate resources to most effectively reach populations of interest. 

Additionally, in our experience, small communities have had difficulty using national projections in their localities. In this process, the national population is generalized into small, “stereotypical” subgroups and detailed projections are made about individuals in target areas. A public health affiliate in a small community may have difficulty in accepting these projections because they may be seen as offensive, broad, or contradictory to current beliefs or understandings about the community. These communities are not likely to use national market research in efforts to improve their programs. 

Planners in Illinois public health programs will continue to use Nielsen community profiles to create and to improve chronic disease prevention and control. These community profiles will be expanded to include more components as additional use of data becomes helpful and new data become available.
